# MiR-204-5p may regulate oxidative stress in myopia

**DOI:** 10.1038/s41598-024-60688-1

**Published:** 2024-04-29

**Authors:** Bo Jiang, Nan Hong, Dongyu Guo, Jianqin Shen, Xilin Qian, Feng Dong

**Affiliations:** 1 Department of Ophthalmology, The First Affiliated Hospital, Zhejiang University School of Medicine, 79 Qingchun Rd., Shangcheng District, Hangzhou, 310003 Zhejiang People’s Republic of China; 2 Department of Clinical Medicine, Capital Medical University, Beijing, China

**Keywords:** MiR-204a-5p, Myopia, Retina, TXNIP, Oxidative stress, Genetics, Molecular biology, Biomarkers, Diseases, Molecular medicine

## Abstract

The mechanisms underlying myopia remain not fully understood. We proposed to examine the function and underlying mechanisms of miR-204-5p in myopia development. The miR-204-5p expression level was assessed in the vitreous humor (VH) of a cohort consisting of 11 patients with high myopia (HM) and 16 control patients undergoing vitrectomy. Then the functional implications of miR-204-5p in ARPE-19 cells were assessed. Thioredoxin-interacting protein (TXNIP) was found as a possible target of miR-204-5p through mRNA sequencing, and its interaction with miR-204-5p was confirmed employing luciferase assay and western blotting. Furthermore, the miR-204-5p function in regulating oxidative stress was examined by measuring reactive oxygen species (ROS) accumulation. The results indicated a significant reduction of miR-204-5p in the VH of HM patients. Overexpression of miR-204-5p suppressed cell proliferation, migration, invasion, and apoptosis in ARPE-19 cells. The direct targeting of miR-204-5p on TXNIP has been confirmed, and its downregulation mediated the miR-204-5p impacts on ARPE-19 cells. Moreover, miR-204-5p overexpression significantly reduced ROS accumulation by targeting TXNIP. Our findings revealed the possible contribution of the miR-204-5p/TXNIP axis in myopia development by regulating oxidative stress, which may provide new targets to combat this prevalent and debilitating condition.

## Introduction

Myopia, also known as nearsightedness, is a common refractive error that affects a large proportion of the worldwide populace. The prevalence of myopia has exhibited a consistent upward trend in recent decades, with a notable concentration in East and Southeast Asia, where as many as 90% of young adults are impacted, with a 10–20% prevalence of high myopia (HM)^1^. HM is known as an axial length (AL) > 26 mm or a minimum refractive error of -6 diopters (D). It is not only a visual impairment but is also associated with several ocular comorbidities, such as glaucoma, cataracts, retinal detachment, and maculopathy, which can result in permanent vision loss^2^. The precise mechanisms involved in myopia development remain incompletely elucidated, and there is a pressing need for further elucidation of its pathogenesis.

Recent research has highlighted the importance of epigenetic modifications in the development of myopia^3–5^. microRNAs (miRNAs) exert control over gene expression through their interaction with the 3’ untranslated region (UTR) of target (messenger RNAs) mRNAs, causing a suppression of translation or mRNA degradation. miRNAs have been implicated in various biological processes, and their dysregulation has been linked to numerous diseases. Several investigations have examined the function of miRNAs in myopia, and we systematically summarized the relevant previous studies^6,7^. For example, a study by Mei et al. identified an upregulation of eight miRNAs in the retina of form-deprived myopic mice, which may function by disturbing their enriched pathways or biological processes^8^. In another study by Guo et al.^9^, a total of 27 miRNAs exhibiting differential expression were identified within the sclera of guinea pigs with lens-induced myopia. Notably, the involvement of the PPAR signaling pathway, as well as the pyruvate and propanoate metabolism pathways, was observed, suggesting their potential contributions to the progression of myopia. Additionally, many studies have investigated the miRNAs function in human myopia. You et al. identified that vitreous miR-145-5p and miR-143-3p are potentially involved in myopic maculopathy development and relate to insulin resistance^10^. Zhu et al.^5^ revealed that the MiR-29a expression was elevated in the aqueous humor of individuals diagnosed with myopia. Furthermore, in vitro experiments demonstrated that MiR-29a effectively suppressed the production of type I collagen in human scleral fibroblast cells.

MiR-204 is considered to be among the most significant miRNAs during eye development in vertebrates and has been implicated in various ocular diseases and biological processes, such as retinal development, lens differentiation, and intraocular pressure regulation^11–13^. Furthermore, our previous study and other researchers’ studies demonstrated that *the PAX6* gene was revealed as a susceptibility gene for HM^14,15^, and studies have shown that *the PAX6* gene controlled the down-regulation of multiple genes through direct up-regulation of miR-204^16^. Nevertheless, the potential impact of miR-204 on myopia development has yet to be investigated.

Vitreous humor (VH) adjacent to the retina serves as an ideal and available material to investigate myopia^5^. The potential significance of retinal pigment epithelium (RPE) cells in the myopia progression has been suggested. The RPE cells have a crucial function in the initiation mechanism of the retina-choroid-sclera complex: the active substance present in the retina serves as the primary signal that interacts with the RPE cells, leading to functional modifications within these cells. Consequently, the RPE cells produce secondary signals that propagate downstream^17^. In this investigation, the initial step involved an examination of the miR-204-5p expression levels in VH specimens from high myopic patients and controls. Then, the miR-204-5p functions were investigated in regulating cell homeostasis and function in RPE cells cultured in vitro. Additionally, we sought to uncover the possible miR-204 target genes and their functional implications in myopia pathogenesis in RPE cells. Our research endeavored to enhance comprehension of the intricate mechanisms underlying myopia development by focusing on the role of miR-204.

## Results

### miR-204-5p expression in high myopic patients

**Figure 1:**
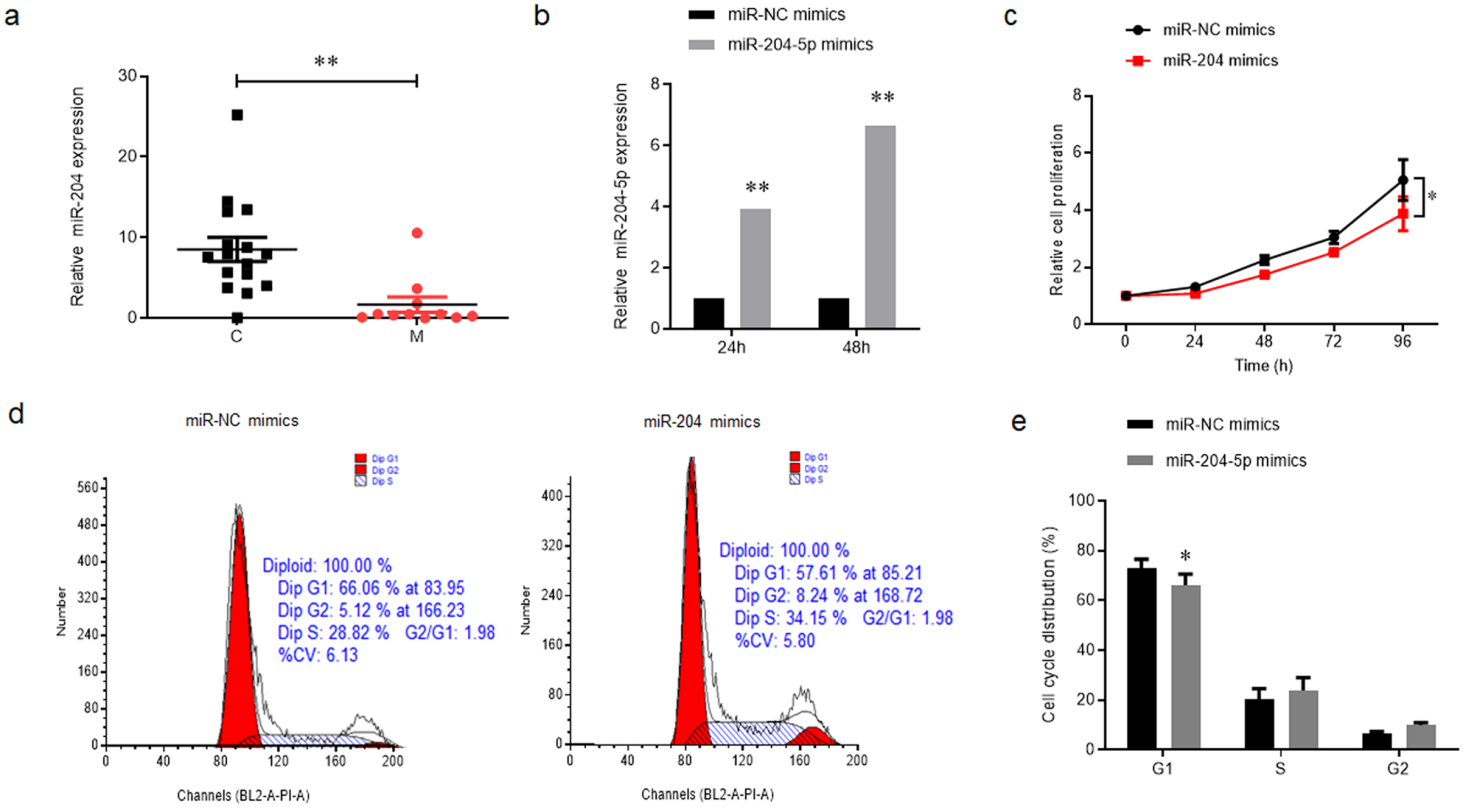
MiR-204-5p was down-regulated in HM and inhibited ARPE-19 cells proliferation. (**a**) The expression levels of miR-204-5p in the VH of patients with HM (M) and controls (C) were examined by qPCR assay. (**b**) ARPE-19 cells were transfected with miR-204-5p mimics or NC mimics for 24 or 48 h, and qPCR assay was used to determine the overexpression efficiency. (**c**) MTT assay was used to compare the cell proliferation activities between the two groups. (**d**) and (**e**) Flow cytometry was used to assess the cell cycle distribution. *P < 0.05; **P < 0.01.

Typically, 27 VH specimens were obtained from individuals who received vitreoretinal surgery. Among these samples, 11 were collected from patients diagnosed with HM, and 16 were obtained from control patients (Table 1). In VH of HM patients, the miR-204-5p expression levels were significantly decreased than that of controls (*p* = 0.001) (Fig. 1a).

**Table 1:** Baseline clinical data of patients (mean ± standard deviation).

Variables	High myopia (n = 11)	Control (n = 16)	*P* value
Age (year)	56.82 ± 3.95 (50 ~ 62)	67.75 ± 5.96 (55 ~ 76)	0.000 95% CI − 15.17 to − 6.70
Sex (male/female)	1/10	5/11	0.189
Axial length (mm)	29.03 ± 1.97	22.92 ± 0.45	0.000 95% CI 4.78 to 7.44
Diagnosis (n)	MH(n = 5), MRS(n = 6)	ERM(n = 9), MH(n = 7)	–

CI: confidence interval.

### miR-204-5p hindered the ARPE-19 cell proliferation

Statistically significant miR-204-5p up-regulation was found at both 24 and 48 h after transfection, and an up-regulation of 6.6-fold was seen after 48 h (*p* = 0.002) (Fig. 1b). Therefore, all subsequent experiments were performed using ARPE-19 cells that were subjected to transfection with miR-204-5p or NC mimics for 48 h. We then assessed if miR-204-5p could have a function in the proliferation of ARPE-19 cells. miR-204-5p effectively inhibited APRE-19 cell proliferation, especially at 96 h time point (*p* = 0.046) (Fig. 1c). To provide additional confirmation if miR-204-5p impaired the cell proliferation by regulating cell cycle distribution, a flow cytometry assay was performed, and the obtained data demonstrated that the count of cells was significantly reduced in the G1 phase upon the introduction of miR-204-5p (*p* = 0.037), while slightly increase the cell proportion in S and G2 phase (Fig. 1d,e).

### miR-204-5p hindered the migration, invasion, and apoptosis of ARPE-19 cells

**Figure 2:**
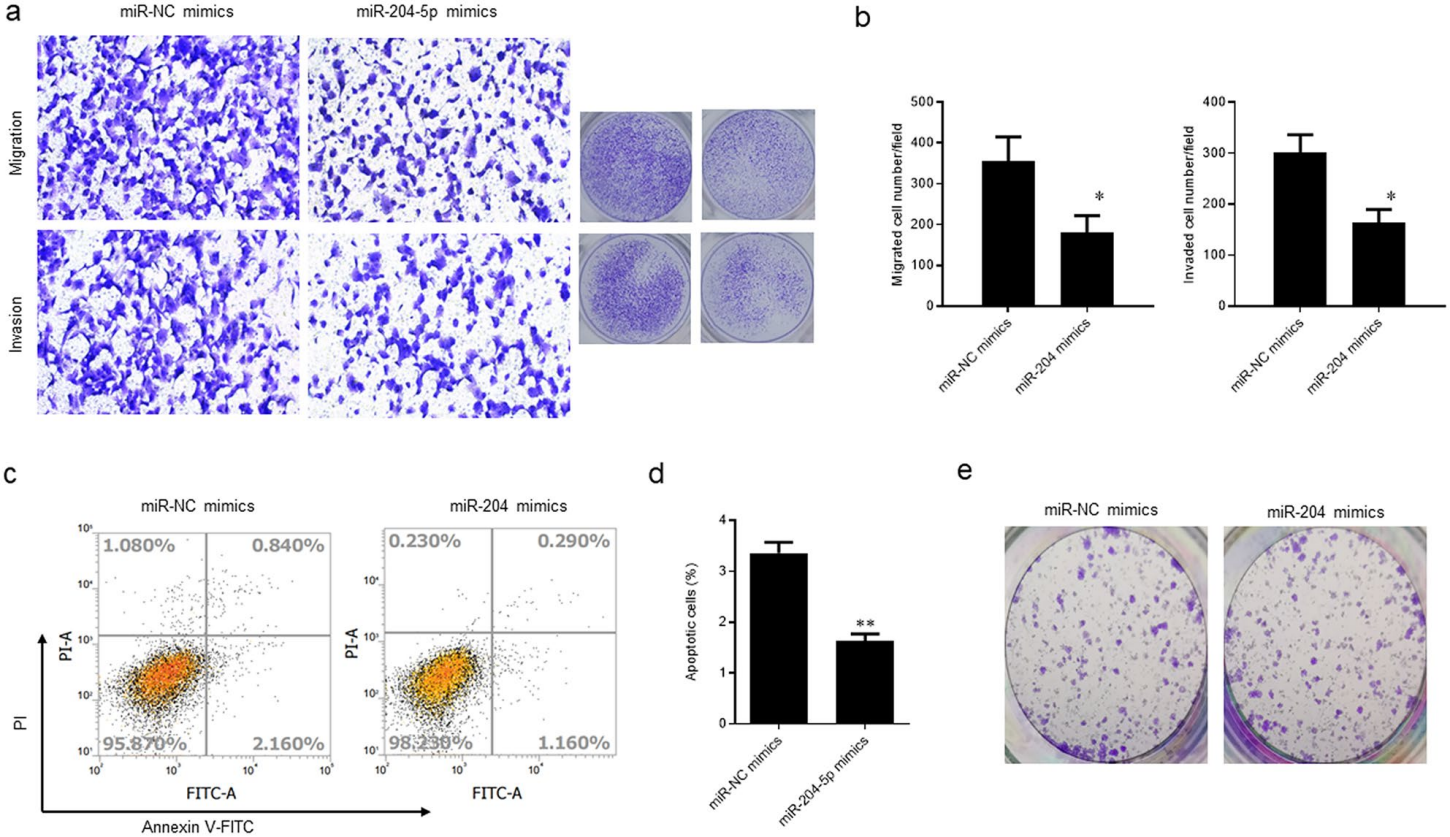
MiR-204-5p suppressed the migration, invasion, and apoptosis of ARPE-19 cells. (**a**) and (**b**) Transwell assays were performed to detect the potentials of cell migration and invasion. (**c**) and (d) Flow cytometry was chosen to assess the apoptosis rate.** e** Plate clone formation assay was used to examine the colony formation activities.

miR-204-5p mimics significantly suppressed both migration activities (*p* = 0.05) and invasion (*p* = 0.04) (Fig. 2a,b). The apoptosis rate of ARPE-19 cells was significantly reduced next to being transfected with miR-204-5p mimics (*p* = 0.003) (Fig. 2c,d). However, no obvious variation was obtained in the colony formation activities between the two groups (Fig. 2e).

### Downstream target gene screening of miR-204-5p

**Figure 3:**
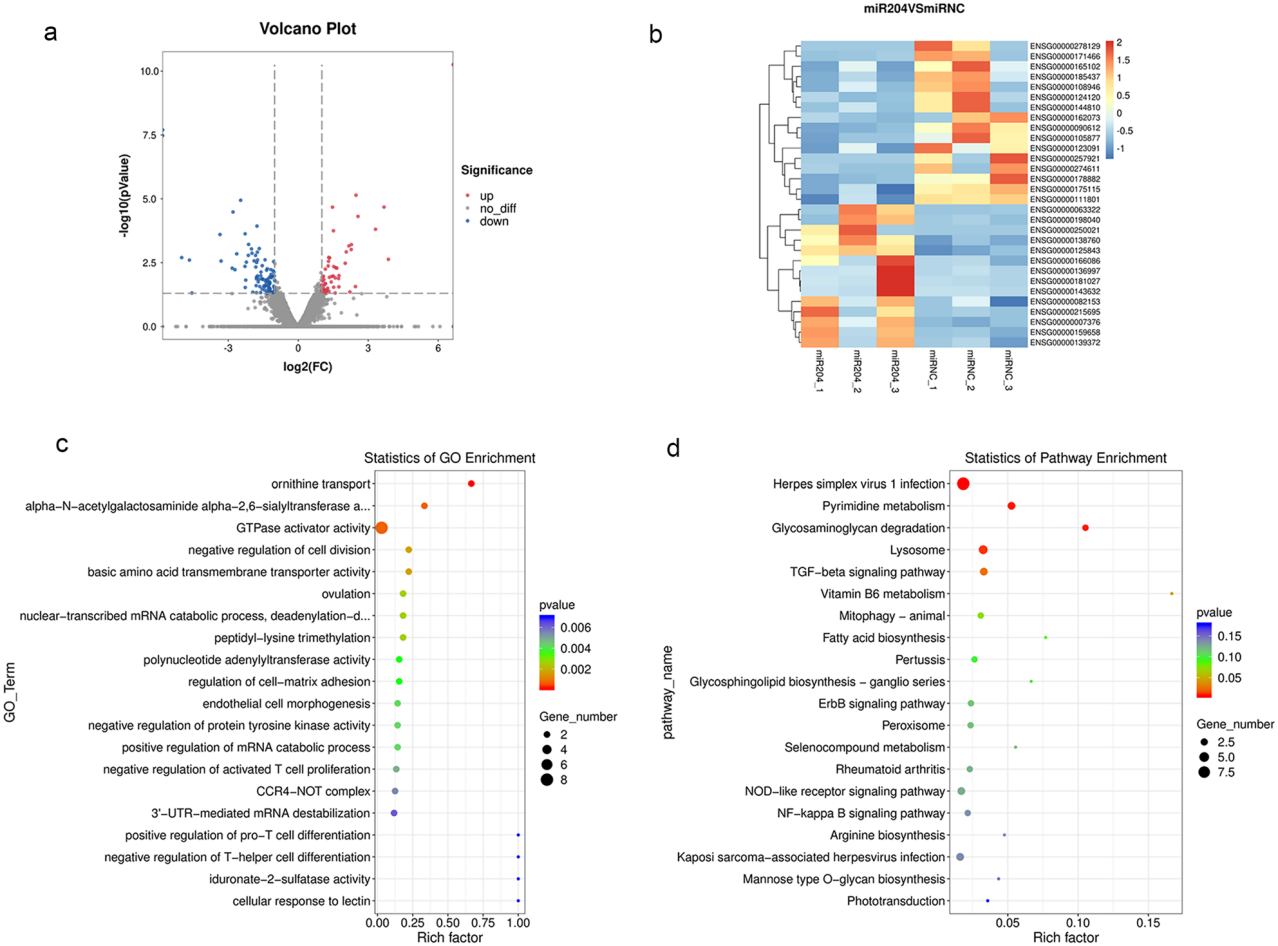
RNAseq in ARPE-19 cells transfected with miR-204-5p or NC mimics. (**a**) Volcano plot for the different expression genes. (**b**) Heatmap for the top 30 different expression genes. (**c**) GO enrichment analysis for all different expression genes. (**d**) KEGG enrichment analysis for all different expression genes.

mRNA sequencing (RNAseq) was employed to achieve a deeper understanding of the mechanisms influencing the regulation of cellular phenotypes by miR-204-5p. RNAseq data revealed there was an up-regulation of 89 genes and a down-regulation of 105 genes in ARPE-19 cells that underwent transfection with miR-204-5p mimics, as contrasted to the NC (Fig. 3a). Figure 3b shows that the heatmap was generated using the top 30 different expression genes. GO and KEGG enrichment analyses of all differential expression genes were also conducted. Figure 3c presents that GO analysis indicated that ornithine transport, alpha−N−acetylgalactosaminide alpha−2,6−sialyltransferase activity, and GTPase activator activity were enriched. The results of KEGG analysis revealed that herpes simplex virus 1 infection, pyrimidine metabolism, glycosaminoglycan (GAG) degradation, lysosome, and TGF—beta signaling pathway were enriched (Fig. 3d).

**Figure 4:**
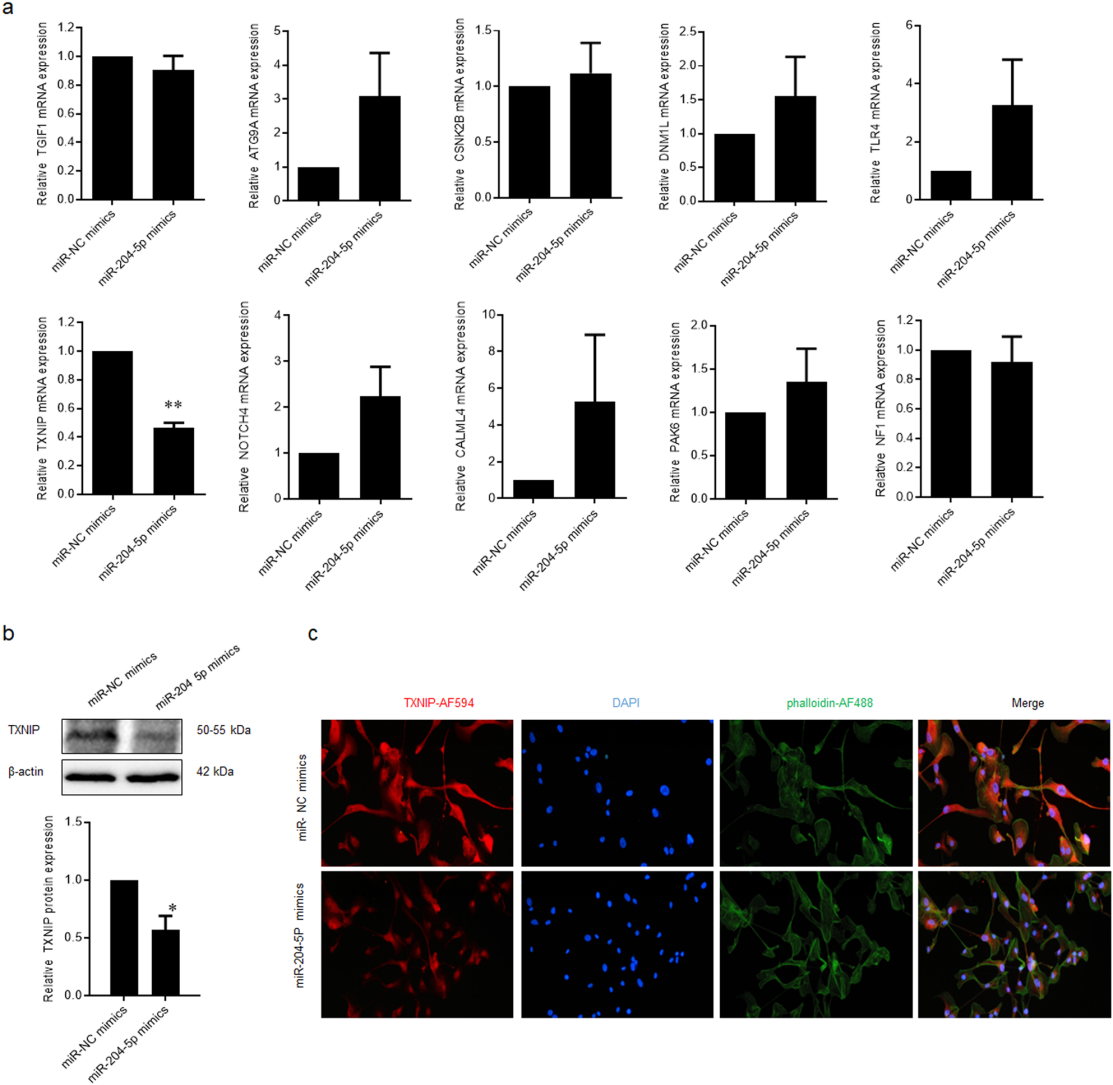
TXNIP was a target gene of miR-204-5p in ARPE-19 cells. (**a**) qPCR was performed to validate the expression profiling of top 10 most significantly differently expressed genes. (**b**) Western blot assay was used to determine the TNXIP protein expression. **c** Immunofluorescence assay was performed to detect the TXNIP protein expression.

Then, the top ten most significantly differentially expressed genes were verified using Quantitative real-time PCR assay (qPCR). Only thioredoxin-interacting protein (TXNIP) mRNA expression was significantly decreased in ARPE-19 cells that underwent transfection with miR-204-5p mimics (*p* = 0.0001) (Fig. 4a). WB assay established that miR-204-5p mimics significantly suppressed the TXNIP protein expression level in ARPE-19 cells (*p* = 0.038) (Fig. 4b). This result was also supported by an immunofluorescence assay (Fig. 4c).

### miR-204-5p influenced the cell proliferation, migration, and invasion by targeting TXNIP

**Figure 5:**
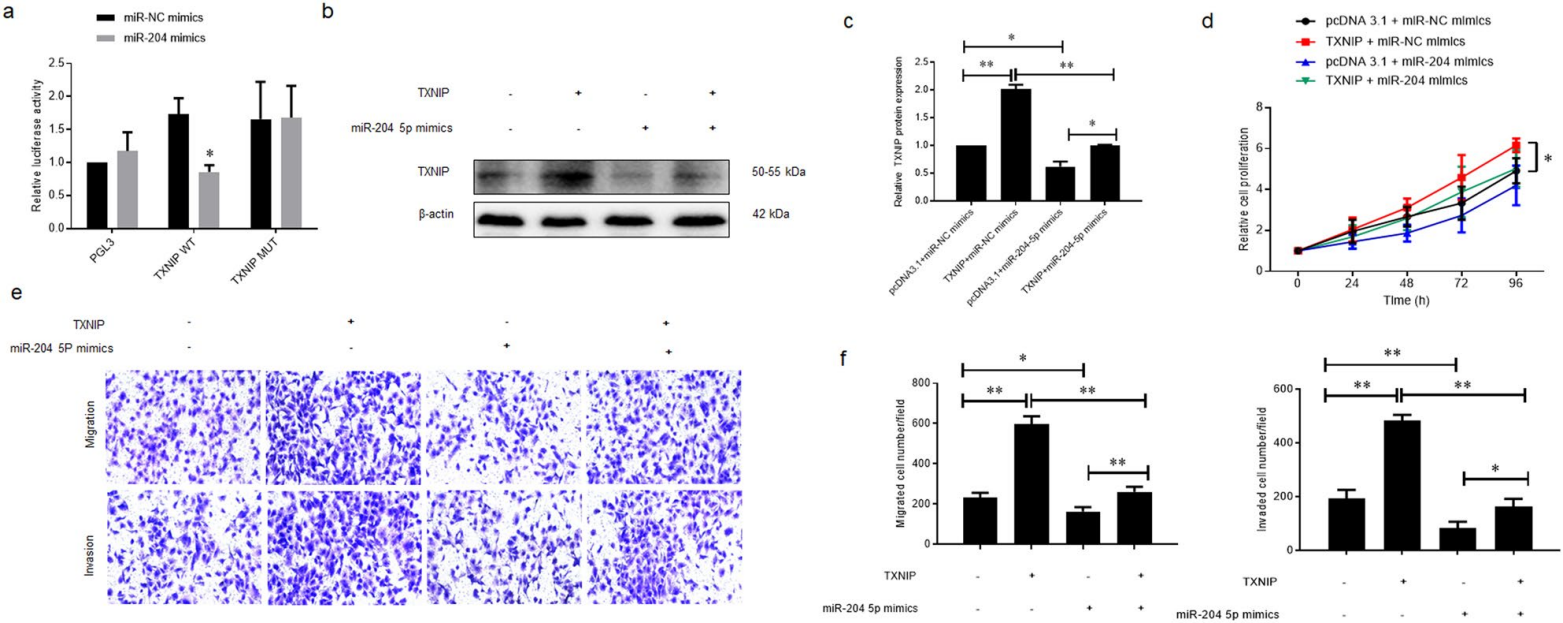
MiR-204-5p influenced the cell proliferation, migration, and invasion of ARPE-19 cells by targeting TXNIP. (**a**) Dual luciferase reporter assay was used to confirm whether miR-204-5p could suppress the TXNIP mRNA expression by directly binding to the potential binding site in its 3’UTR region. (**b**) and (**c**) ARPE-19 cells were transfected with TXNIP overexpression plasmids or/ and miR-204-5p mimics, or the corresponding control for 48 h respectively. Western blot assay was used to examine the expression of TXNIP protein. (**d**) ARPE-19 cells were treated as in B, MTT assay was used to detect the cell proliferation. (**e**) and (**f**) ARPE-19 cells were treated as in B, Transwell assays were performed to examine the cell migration and invasion potentials.

Dual-luciferase Reporter Assay (DLRA) was conducted to further validate the direct targeting of TXNIP by miR-204-5p. miR-204-5p mimics could significantly decrease the luciferase activity of WT plasmid (p = 0.004) (Fig. 5a) while failing in the MUT one. WB assay also showed that TXNIP protein was overexpressed in ARPE-19 cells after transfecting with TXNIP overexpression plasmid (*p* = 0.003), while co-transfection with miR-204-5p mimics effectively reduced its expression level (*p* = 0.003) (Fig. 5b,c). TXNIP overexpression not only promoted cell proliferation but also effectively counteracted the inhibitory impact mediated by miR-204-5p (*p* = 0.036) (Fig. 5d). TXNIP overexpression also effectively counteracted the inhibitory impact mediated by miR-204-5p in the Transwell assays (migration: *p* = 0.0003; invasion: *p* < 0.0001) (Fig. 5e,f).

### miR-204-5p regulates oxidant stress by targeting TXNIP

**Figure 6:**
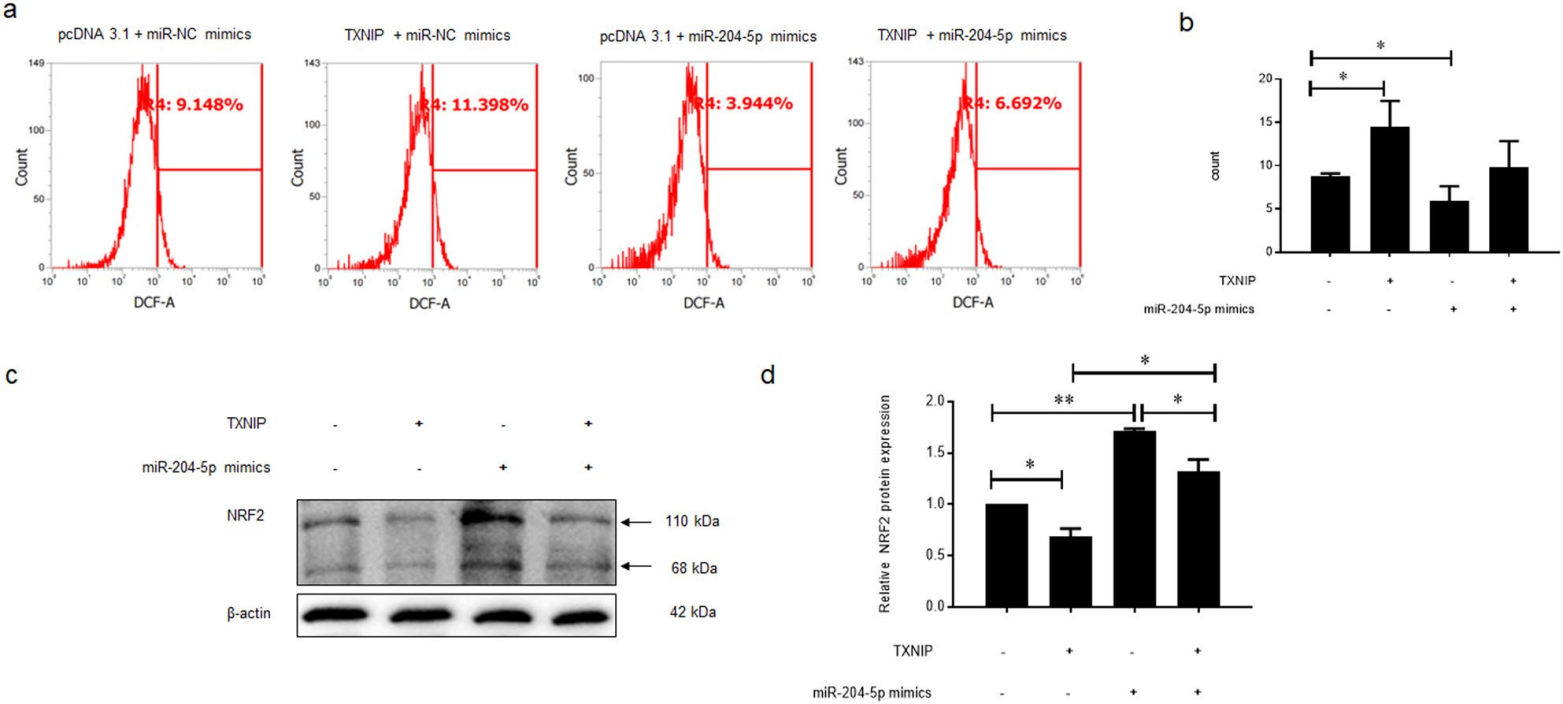
MiR-204-5p regulates oxidant stress by targeting TXNIP in ARPE-19 cells. (**a**) and (**b**) Flow cytometry assay was used to test the ROS accumulation. (**c**) and (**d**) Western blot assay was chosen to detect the protein expression of NRF2.

Previously published research has indicated that TXNIP could promote oxidative stress by inhibiting the anti-oxidative function of thioredoxin (TRX)^18^. miR-204-5p mimics significantly decreased ROS accumulation (*p* = 0.033), while TXNIP exerted an opposite impact (*p* = 0.047) (Fig. 6a,b). It is also well known that NRF2 could be activated and function as a negative regulator of oxidant stress^19^. WB assay illustrated that miR-204-5p mimics effectively promoted the NRF2 protein expression (*p* = 0.0003), while TXNIP not only dramatically down-regulated NRF2 (*p* = 0.03), but also impaired the miR-204-5p mimics function (*p* = 0.04) (Fig. 6c,d).

## Discussion

The present investigation proposed to examine the miR-204-5p expression and function in myopia and correlated retinopathy. The current investigation illustrated that miR-204-5p was hindered in the VH of eyes with HM and could suppress proliferation and apoptosis and suppress migration and invasion of ARPE-19 cells. The bioinformatics analysis suggests that miR-204-5p has the ability to modulate genes involved in myopia-related pathways. RNAseq was done, and only TXNIP mRNA downregulation in ARPE-19 cells was verified by qPCR. Moreover, our outcomes revealed that the TXNIP expression was decreased in RPE cells via miR-204-5p overexpression, which indicated that increased miR-204-5p expression could downregulate TXNIP expression. Finally, our outcomes revealed that miR-204-5p downregulation may increase TXNIP expression and contribute to myopia development via enhanced oxidant stress. These findings offer persuasive evidence about the significant role played by miR-204-5p in the modulation of retinal cell behavior and myopia development.

MiR-204 is located within intron 6 of the TRPM3 gene, situated on chromosome 9q21.12 within the human genome^13^. The seed regions of miR-204 exhibit a high degree of conservation across species, such as humans and mice, indicating that miR‐204 may have an essential regulatory role in the evolutionarily conserved gene expression. It is evident that miR-204 exhibits diverse functions in the modulation of gene expression within ocular tissues. Mutation in miR-204 was first found in a large family with varying degrees of retinal dystrophy and bilateral coloboma^20^. Multiple types of cataracts are associated with differential regulation of miR-204 in mice and humans^13^. MiR-204 was also observed to be downregulated in glaucomatous rat retina, influencing TGF-β signaling^12^. In this investigation, we identified a novel finding that miR-204-5p was decreased in the VH of eyes with HM.

Prior research indicated that miR-204 is involved in the phenotype preservation, differentiation of the RPE, and maturation of adjacent photoreceptors. Wang et al. showed that miR-204 exhibits significant upregulation in the human fetal RPE and has an essential function in the preservation of epithelial barrier function and cellular physiology^21^. MiR-204 facilitates the differentiation and maturation of RPE cells and adjacent photoreceptor cells by targeting genes associated with the epithelial-to-mesenchymal transition (TGFBR2) and the Wnt/β-catenin pathway^13^. In this investigation, we found that the RPE proliferation was suppressed by miR-204-5p by induction of G1 phase arrest.

Bioinformatics analysis displayed that the GAG degradation pathway, lysosome, and TGF − beta signaling pathway had significant enrichment in the KEGG pathway of putative target genes. Insulin affects both the elongation of the eye and the thickness of the choroid to modulate myopia development. Sheng et al. found that insulin leads the RPE to create signal molecules and increase scleral GAG synthesis through choroid, an eye growth indicator^22^. Zhang et al. conducted in vitro and in vivo experiments, which provided evidence supporting the notion that elevated levels of miR-204 in RPE can potentially delay the onset of diseases by inhibiting the generation of oxidative stress and inflammation. Furthermore, their findings suggest that the regulation of autophagy and endolysosomal interaction by miR-204 in RPE has a crucial function in preserving the normal structure and function of the RPE and retina^23^. The TGF-β superfamily primarily participates in the modulation of scleral remodeling in myopia. Chen et al. demonstrated that there was an increase in TGF-β2 activity in the sclera of guinea pigs with lens-induced myopia^24^. The gradual reduction in the collagen I expression throughout the process of myopic sclera remodeling indicates a potential involvement of TGF-β1 signaling in myopia development and progression^25^. The current bioinformatics analysis indicated that miR-204 target genes were involved in myopia-related pathways and affect retinal structure and function.

TXNIP is a multifunctional adaptor protein belonging to the α-arrestin protein family, which is a negative regulator of TRX function and promotes cellular inflammation, oxidative stress, and premature cell death^26^. Yumnamcha et al. discovered that the TXNIP-Trx-TrxR redox pathway potentially plays a role in the impairment of RPE function in diabetic retinopathy as well as other neurodegenerative diseases affecting the retina^27^. Microarray analysis of cataracts suggested that miR-204 downregulation was linked to TXNIP up-regulation, while similar anti-oxidative genes were upregulated or downregulated^28^. In our study, TXNIP overexpression promoted the proliferation of RPE cells and significantly reversed the inhibitory impact mediated by miR-204-5p. Since miR-204-5p expression was down-regulated in patients with HM, it was reasonable to hypothesize that miR-204-5p downregulation promotes TXNIP expression, resulting in cytotoxicity from oxidant stress in RPE cells.

There is a close relationship between oxidative stress and myopia, both in terms of development and treatment. Wu et al. established the fundamental significance of hypoxia in the process of scleral ECM remodeling and the development of myopia^29^. Higher ROS generation can lead to cellular damage and inflammation, and retina damage induced by oxidative stress has been associated with hypoxia^30^. Recent metabolomic studies of VH and blood serum in myopic patients have indicated that the metabolic pathways of myopia samples are closely related to oxidative stress^31,32^. Anti-oxidants are shown to improve collagen fracture and fiber arrangement, suggesting that lowering oxidative stress may reduce the development of myopia, possibly secondary to chronic scleral inflammation^33^. The findings of the current investigation align with those of prior studies. We observed that the miR-204-5p overexpression significantly hindered the accumulation of ROS and promoted the NRF2 expression in RPE cells. This result also established that miR-204-5p might play a role in myopia development through the regulation of oxidative stress. It is hypothesized that miR-204-5p downregulation promotes TXNIP expression and may contribute to myopia development by inducing cellular oxidative stress and disrupting retinal cell homeostasis and function while down-regulating anti-oxidative genes (e.g., NRF2) expression.

One of the limitations of this study was the small number of cases. Although a statistical difference in miR-204-5p expression was observed in the HM samples and the number of participants was similar to that reported in recent literature^5,10^, further studies may consider expanding the sample size and stratifying analyses by variables such as the grade of myopic maculopathy. Additionally, our study only focused on the regulation of miR-204-5p through TXNIP, and further animal experiments s are warranted to investigate the crosstalk between miR-204-5p and other regulatory molecules or signaling pathways involved in myopia development.

In conclusion, our study provided a novel perspective into the miR-204-5p function in myopia and highlighted the possible involvement of the miR-204-5p-TXNIP axis in the modulation of retinal cell behavior and oxidative stress. These findings contributed to our comprehension of the intricate molecular mechanisms that underlie myopia development. Moreover, these outcomes have significant consequences for the discovery of novel biomarkers and the advancement of targeted therapeutic approaches for the treatment of myopia. Future studies should focus on elucidating the precise network of mechanisms through which miR-204-5p modulate retinal cell function and oxidative stress.

## Methods

### Patient and specimen collection

The VH specimens were acquired from individuals who underwent surgical procedures for idiopathic epiretinal membrane (ERM), myopic retinoschisis (MRS), and macular hole (MH) at the 1st Affiliated Hospital, Zhejiang University School of Medicine, during the period spanning from March 2020 to December 2021. The HM group encompassed surgically treated eyes with AL > 26.0 mm. The control cohort comprised eyes that had undergone surgical treatment, with AL ranging from 22.0 to 24.0 mm. All participants in the study were adult individuals who received thorough preoperative ophthalmic assessments. These assessments encompassed various diagnostic procedures such as visual acuity testing, indirect ophthalmoscopy, slit-lamp biomicroscopy, fundus photography, optical coherence tomography, B-scan ultrasonography, and AL measurements using an optical biometer (OA-2000, Tomey, Nagoya, Japan). The exclusion criteria encompassed a history of ocular disease other than mild cataract, including glaucoma, vascular disease, age-related macular degeneration, previous ocular trauma, history of intravitreal injection or intraocular surgery, systemic diseases with known ocular involvement, or other factors that could potentially disrupt the composition of VH. Table 1 provides a concise summary of the baseline information pertaining to the patients. During the initiation of the vitrectomy procedure, a volume of 0.5–1.0 mL of undiluted vitreous sample was extracted through the vitrectomy probe and collected in a sterile 5-mL syringe that was connected to it. The administration of a saline solution into the vitreous cavity was promptly initiated in order to restore the intraocular pressure of the eye. The VH samples were rapidly frozen and subsequently stored at a temperature of − 80 °C until they were ready for subsequent use. This project was approved by the Ethics Committee of the 1st Affiliated Hospital, Zhejiang University School of Medicine and was conducted in accordance with the Declaration of Helsinki. All participants provided written informed consent for the use of these clinical materials in research.

### Quantitative real-time PCR assay (qPCR)

The RNA from VH was purified employing an RNeasy Micro Kit (Qiagen) in line with the guidelines of the manufacturer. The extraction of total RNA from cell samples was performed by Trizol reagent (Invitrogen). The first-strand cDNA was obtained utilizing M-MLV reverse transcriptase (Promega) supplemented with dNTP reagents (Invitrogen). An ABI 7500 Real-Time PCR system (Thermo Fisher Scientific) was employed to conduct the PCR reaction in line with the manufacturer’s instructions with 2X Power SYBR Green PCR Master Mix (Thermo Fisher Scientific). The PCR reaction procedure commenced with an initial denaturation phase at 95 °C, which lasted for 10 min. Subsequently, a series of 40 cycles was conducted, wherein each cycle encompassed a denaturation phase at 95 °C for 15 s, succeeded by an annealing and extension phase at 52 °C for 1 min. The relative RNA expression was measured by the 2^−ΔΔCt^ method utilizing β-actin (for mRNA) or U6 for (miRNA) as control. Table S1 presents the primer sequences for qPCR.

### Cell culture

The ARPE-19 cell line, derived from human RPE, was acquired from the Chinese Academy of Sciences. The cells were cultivated in DMEM (Gibco), which was enriched with 10% inactivated fetal bovine serum (FBS) (Gibco). The culture was maintained within a controlled environment of a humidity-controlled incubator supplemented with 5% CO_2_ at 37 °C. The regular cell subculture was conducted using a 0.05% trypsin solution, and all experiments were conducted with cells derived from passages 3–15, ensuring a minimum of three repetitions.

### Plasmid construction and transfection

The coding sequence of human TXNIP mRNA was manufactured and introduced into the pcDNA3.1 vector to develop the TXNIP overexpression plasmid. The plasmid’s integrity was confirmed through the utilization of DNA sequencing. The miR-204-5p mimics and their negative control (NC) mimics were acquired from GenePharma (Shanghai, China). All transfections for both overexpression plasmid and miRNA mimics were conducted employing Lipofectamine 2000 reagents (Invitrogen, Carlsbad, CA) in line with the protocol of the manufacturer. Following transfection, the expression effectiveness was validated by qPCR or western blot (WB) assays.

### MTT assay for cell proliferation

The ARPE-19 cell proliferation potential with miR-204-5p overexpression or co-transfected with TXNIP overexpression plasmid was evaluated by the methyl tetrazolium (MTT) assay. A total of 5000 ARPE-19 cells were introduced into a 96-well plate and given sufficient time to adhere overnight. Then, the cells were subjected to transfection with miR-204-5p mimics or/and TXNIP overexpression plasmid or their controls, respectively, for 48 h. Following transfection, the supernatant was eliminated, and fresh medium was added and incubated for another 24, 48, 72, and 96 h. At the conclusion of each time interval, the medium was discarded, and MTT solution (1 mg/mL, 50 μL) was introduced. The samples were subsequently incubated at 37 °C for a maximum duration of 4 h. Following this incubation period, to dissolve the purple crystals, a volume of 150 μL of DMSO was introduced to each well. The measurement of absorbance at OD570 nm was performed employing a microplate reader (Molecular Device).

### Flow cytometry assay for cell cycle distribution, apoptosis, and reactive oxygen species (ROS) level

The ARPE-19 cells were subjected to a 24-h incubation period in DMEM supplemented with 0.1% FBS for cell cycle synchronization. After that, the cells were subjected to transfection with miR-204-5p mimics or NC mimics for 48 h, and then adding fresh DMEM with 10% FBS was performed. After 24 h incubation, cells were collected, subjected to a PBS wash, and subsequently fixed in 75% ethanol for an overnight duration. Subsequently, the cells underwent a second round of washing with PBS. Following this, they were subjected to incubation with RNase A at a concentration of 100 μg/mL and 0.1% Triton X-100 for 0.5 h at 37 °C. Afterward, the cells were subjected to staining with propidium iodide (PI) at 30 μg/mL for a period of 10 min at 37 °C in a dark environment. The cell cycle distribution was assessed by employing the Attune NxT Flow Cytometer (ThermoFischer). The FlowJo software program was utilized to calculate the cell proportions in the G1, S, and G2/M stages.

The FITC Annexin V Apoptosis detection kit (BD Biosciences) was employed to assess the ARPE-19 apoptosis rate cells subsequent to the miR-204-5p overexpression. In this experiment, ARPE-19 cells were initially placed into a six-well plate and left to incubate overnight to facilitate cell attachment. Subsequently, the cell transfection was conducted with either miR-204-5p mimics or NC mimics for a duration of 48 h. Both suspension and adjacent cells were gathered and rinsed with chilled PBS. Subsequently, the cell resuspension was conducted in 0.5 mL of binding buffer and subjected to incubation with 5 μL of Annexin V-FITC and 5 μL of PI for a duration of 15 min at room temperature (RT) while maintaining a dark environment. The determination of cell apoptosis rates was conducted using the Attune NxT Flow Cytometer.

To detect ROS levels, after ARPE-19 cells were treated, cells were collected, and a single-cell suspension was acquired by pipetting up and down gently. Cells were subjected to incubation with 20 μM DCFDA in FBS-free DMEM for 30 min at RT and then analyzed by Attune NxT Flow Cytometer.

### Transwell assays for cell migration and invasion

Transwell assay was deployed for the assessment of migration and invasion of cells. ARPE-19 cells were cultivated in 6-well plates in media containing DMEM with 10% FBS overnight and serum-starved for 24 h. After that, the cell transfection was performed with miR-204-5p mimics or NC mimics for a duration of 48 h. Following that, the cells were gathered, rinsed with chilled PBS, and reconstituted in DMEM devoid of FBS. A 100 μL cell suspension (3 × 10^4^ cells) was introduced to the top chamber of the transwell, and the lower section was plated into a 24-well plate in DMEM with 10% FBS.

Cells were allowed to culture for 24 h, and a cotton swab was employed to eliminate non-migrated cells through the filter. Migrated cells on the bottom filter were immobilized with iced absolute methanal for 15 min, stained with 0.5% crystal violet-stained for 10 min at 37 °C, respectively, followed by counting under a microscope (Olympus). Three views were taken for each well and used for cell counting. The average results were calculated from three independent experiments. For cell invasion, prior to cell seeding, the Transwell filters were pre-coated with Matrigel. All other steps were consistent with those in the migration assay.

### Colony formation assay

The transfection of ARPE-19 cells was performed with miR-204-5p mimics or NC mimics for a duration of 48 h. Subsequently, 500 cells from each group were cultivated into individual wells of a six-well plate and incubated for a period of 14 days. Subsequently, the cell washing was performed with chilled PBS, followed by treatment with methanol at 37 °C for 15 min in order to achieve fixation. Following incubation at 37 °C for 10 min in PBS, the specimens were exposed to staining using a 0.1% solution of crystal violet. Subsequently, the colonies that exhibited a minimum of 50 cells were enumerated using a microscope (Olympus).

### mRNA sequencing and bioinformatics analysis

To further explore the underlying mechanisms through which miR-204-5p exerted its biological roles in ARPE-19 cells. The mRNA expression profiling was performed using RNAseq in ARPE-19 cells that underwent transfection with miR-204-5p mimics or NC mimics for 48 h. The extraction of total RNA from cell samples was carried out employing a Trizol reagent (Invitrogen). RNAseq was conducted by Novogene (Beijing, China). The DESeq2 R package (version 1.20.0) was utilized to acquire various expressed genes. Subsequently, the clusterProfiler R package (version 3.8.1) was employed to conduct GO and KEGG pathways enrichment analysis on the differentially expressed genes. The DESeq2 package offers statistical algorithms for identifying differential expressions within digital gene expression datasets by employing a model that is dependent on the negative binomial distribution. The obtained P-values were subjected to adjustment employing the Benjamini and Hochberg method, which is commonly employed to regulate the false discovery rate. The threshold for significantly differential expression was established as padj < 0.05 and |log2(foldchange)|> 1.

#### Luciferase reporter assay

The potential miR-204-5p binding site in the 3’UTR of TXNIP mRNA was obtained using TargetScan and miRTarBase datasets. Next, the 3’UTR fragments of TXNIP mRNA comprising the miR-204-5p binding site (WT) and mutant binding site (MUT) were subcloned into pmirGLO vectors, respectively (Promega, Madison, USA). The co-transfection of pmirGLO-TXNIP–3′UTR (WT/MUT) plasmid with miR-204-5p mimics or NC mimics was performed into ARPE-19 cells for 48 h. The transfection procedure involved the introduction of a CMV-renilla plasmid into all cells, serving as an internal control. The measurement of luciferase activity was conducted employing a DLRA Kit (Promega, Madison, USA) per the guidelines of the manufacturer.

#### WB assay

Following the rinsing of the cells with PBS, the proteins were acquired through the lysis of the cells in a chilled solution of RIPA buffer, which was further enriched with a mixture of protease and phosphatase inhibitors (Sigma) for a duration of 10 min. The supernatant was obtained through centrifuging at a force of 14,000 × g for 15 min at 4 °C. The protein level in the supernatant was assessed by deploying a BCA kit (Thermofisher). From each sample, 20 μg of protein was separated by SDS-PAGE for 60 min at an applied voltage of 200 V. Afterward, transferring the separated proteins was conducted onto a PVDF membrane. The membranes were obstructed using a solution of 5% fat-free milk in PBS enriched with 0.1% Tween-20 (PBST). Following the washing step with PBST, the membranes were subjected to incubation with the primary antibodies (TXINIP from Abcam and NRF2 from CST), which were appropriately diluted at a ratio of 1:1000 in TBST containing 5% BSA. This incubation was carried out overnight at a temperature of 4 °C. Following undergoing three washes in PBST, the membranes were subjected to incubation with horseradish peroxidase-conjugated goat anti-mouse or anti-rabbit IgG (Santa Cruz, diluted at a ratio of 1:5000) for a duration of 4 h. After three washes with PBST, the membrane was soaked with ECL reagents (Thermofisher), and signals were visualized using X-ray film (Kodak). The quantification of band intensity was conducted employing the ImageJ software (NIH, Bethesda, MD, USA). The obtained results were subjected to normalization using β-actin as a reference.

#### Immunofluorescence assay

ARPE-19 cells (1 × 10^4^ cells/well) were seeded on coverslips, which were placed in the 24-well plate in advance and incubated for 24 h. The cells were subjected to transfection with miR-204-5p mimics or NC mimics for 48 h. After the transfection procedure, the cells underwent a series of three washes using PBS and were subsequently fixed with a 4% paraformaldehyde solution for a duration of 1 h. Following the washing step with PBS solution with 0.3% Triton X-100 (PBS-T), the ARPE-19 cells were subjected to a 2-h incubation period with primary antibodies (TXNIP-AF594). Afterward, the cells were exposed to a PBST wash to eliminate any remaining antibodies, followed by 2 h-incubation with phalloidin-AF488 at RT. After nucleus staining by DAPI, fluorescence images were acquired under a fluorescent microscope (Olympus).

#### Statistical analysis

Data were presented as the means ± standard deviations (S.D.) from at least three autonomous trials. Normality of continuous variables was assessed with the Shapiro–Wilk test. Student’s t-test analyzed differences in normally distributed data between groups, while the Mann–Whitney U test was applied to non-normally distributed data (relative miR-204-5p expression data). Fisher’s exact test compared categorical variables between two groups.A *P* value < 0.05 (*) was deemed significant.

### Supplementary Information

Supplementary Table S1.

Supplementary Figures.

## Data Availability

The datasets used and analysed during the current study are available from the corresponding author on reasonable request.
