# Supplementary material for: MiR-204-5p may regulate oxidative stress in myopia
Source: Sci Rep. 2024 Apr 29;14:9770. doi: 10.1038/s41598-024-60688-1 (PMC11059383; doi:10.1038/s41598-024-60688-1)
Supplement: Supplementary file 1 — Supplementary Table S1. [file 41598_2024_60688_MOESM1_ESM.docx]

**Table S1 Primer Sequences for qPCR.**

| **genes** | **F（5’-3’）** | **R（5’-3’）** |
| --- | --- | --- |
| TGIF1 | CTCCTAAGCCGTCATCCC | CCACTGAAGTCCTGGTTG |
| ATG9A | CATGGTGGCACTGGTTAA | CACAGCAGGAAGTTAGCG |
| CSNK2B | GCCGAGATGCTTTATGGA | GGAAACCAGTGCCGAAGT |
| DNM1L | GCTCCAGGACGTCTTCAACA | GTTTTTCCATGTAGCAGGGTCA |
| TLR4 | CAGAAGCTGGTGGCTGTG | ATGTAGAACCCGCAAGTC |
| TXNIP | GTCATCAGTCAGAGGCAATC | CATCTCAGAGCTGGTTCG |
| NOTCH4 | TACAGGCTACACAGGACCCA | CTGTTCACACAGGTACCCCC |
| CALML4 | CAGACCCACGGGATAGAC | TCATACTTCACTTTGCCATT |
| PAK6 | GCAGGCTATTCCGAAGC | CCGAAGTGGTCAGAGGG |
| NF1 | ACTTGCCACTCCCTACTGAA | CCCCTTTCGATTCTAGGTGGT |
| β-actin | AGCAGTTGTAGCTACCCGCCCA | GGCGGGCACGTTGAAGGTCT |
| U6 | CTCGCTTCGGCAGCACA | AACGCTTCACGAATTTGCGT |
| mircro-204 | CGAGGTATTCGCACT | TCCCTTTGTCATCCT |
| mircro-  204 RT | GTCGTATCCAGTGCAGGGTCCGAGGTATTCGCACTGGATACGACAGGCAT | |
