# Supplementary material for: MiR-204-5p may regulate oxidative stress in myopia
Source: Sci Rep. 2024 Apr 29;14:9770. doi: 10.1038/s41598-024-60688-1 (PMC11059383; doi:10.1038/s41598-024-60688-1)
Supplement: Supplementary file 2 — Supplementary Figures. [file 41598_2024_60688_MOESM2_ESM.pptx]

## Slide 1
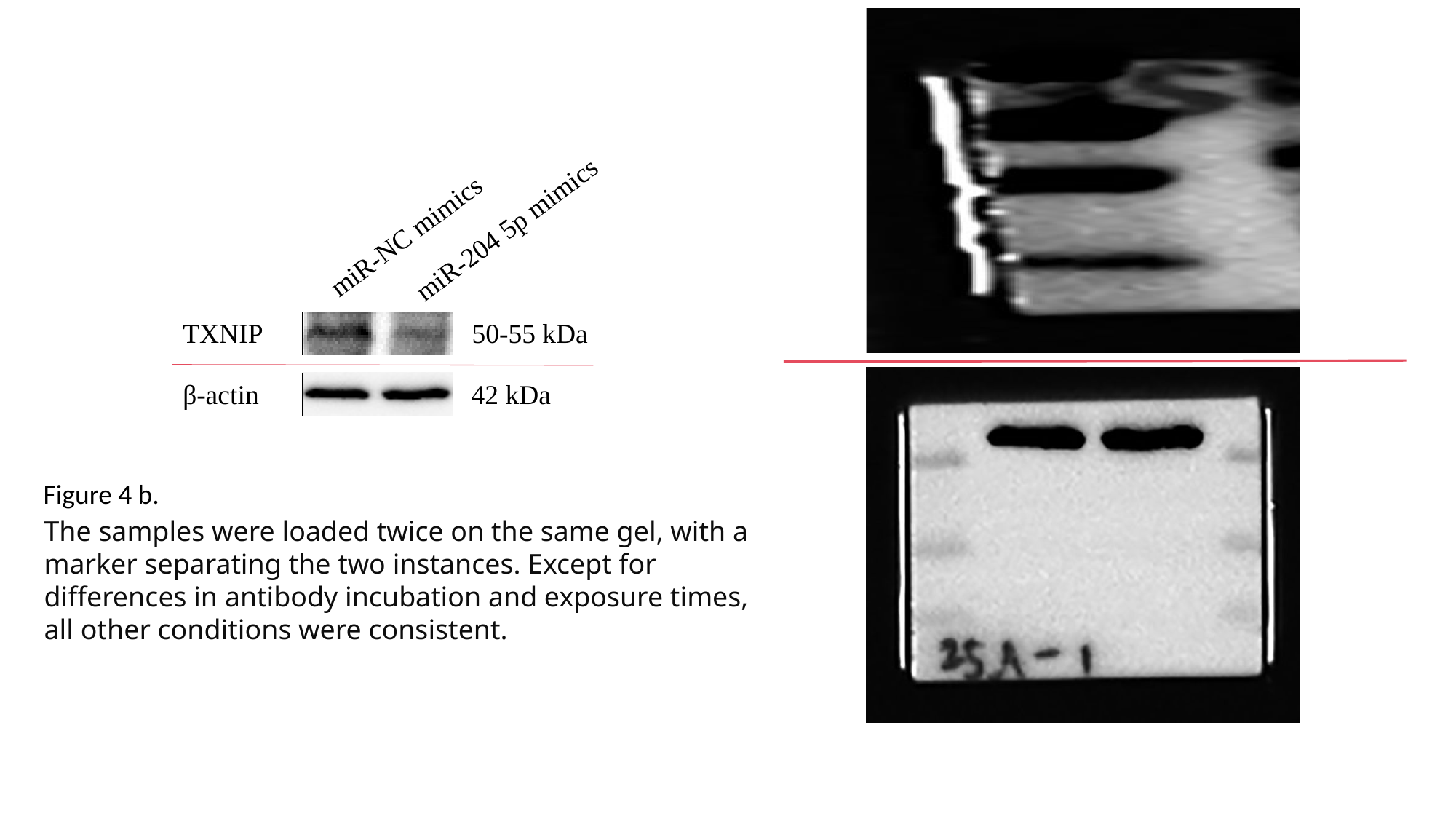

miR-204 5p mimics
miR-NC mimics
TXNIP
50-55 kDa
β-actin
42 kDa
Figure 4 b.
The samples were loaded twice on the same gel, with a marker separating the two instances. Except for differences in antibody incubation and exposure times, all other conditions were consistent.

## Slide 2
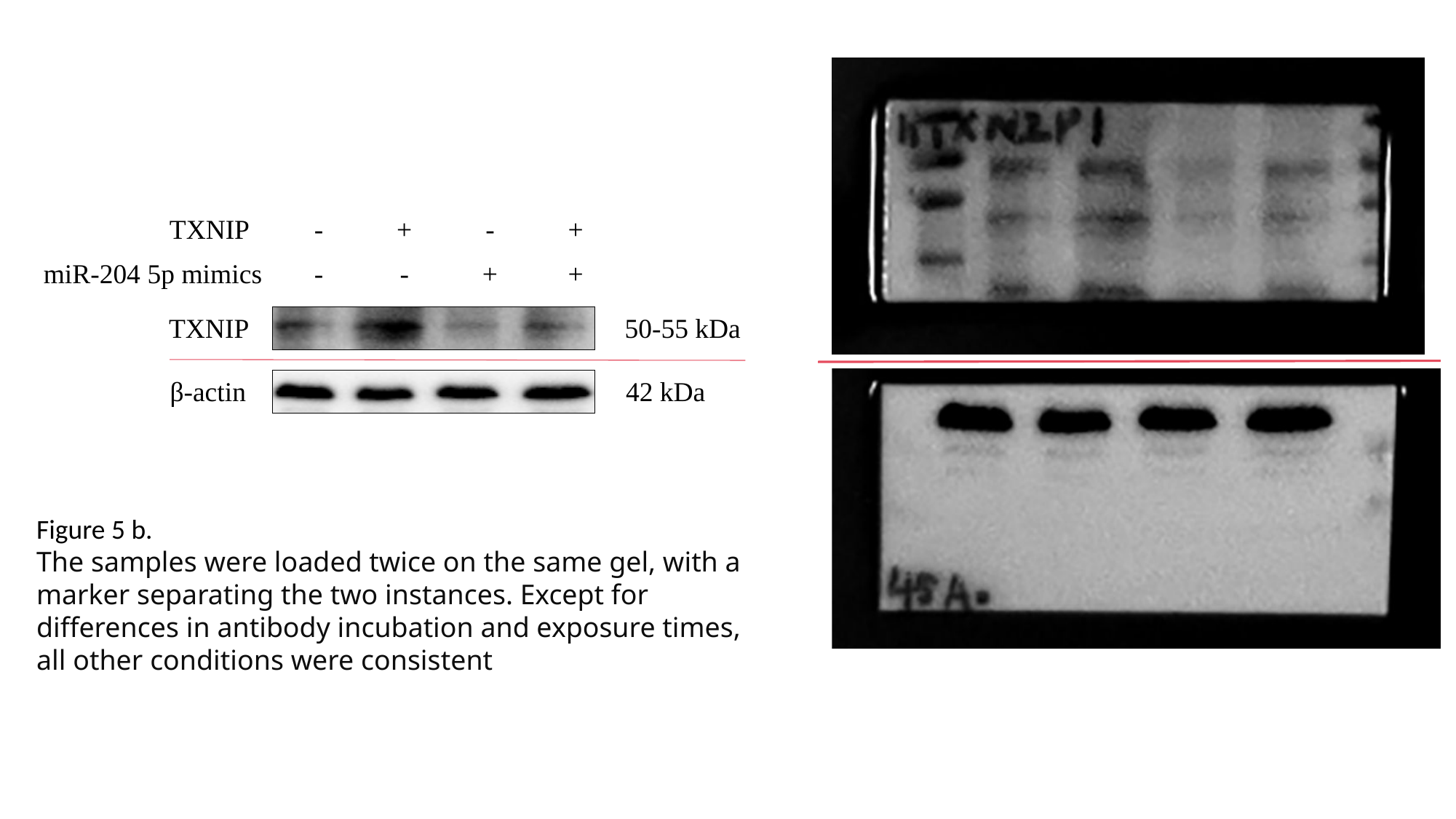

TXNIP
-
+
-
+
miR-204 5p mimics
-
-
+
+
TXNIP
50-55 kDa
β-actin
42 kDa
Figure 5 b.
The samples were loaded twice on the same gel, with a marker separating the two instances. Except for differences in antibody incubation and exposure times, all other conditions were consistent

## Slide 3
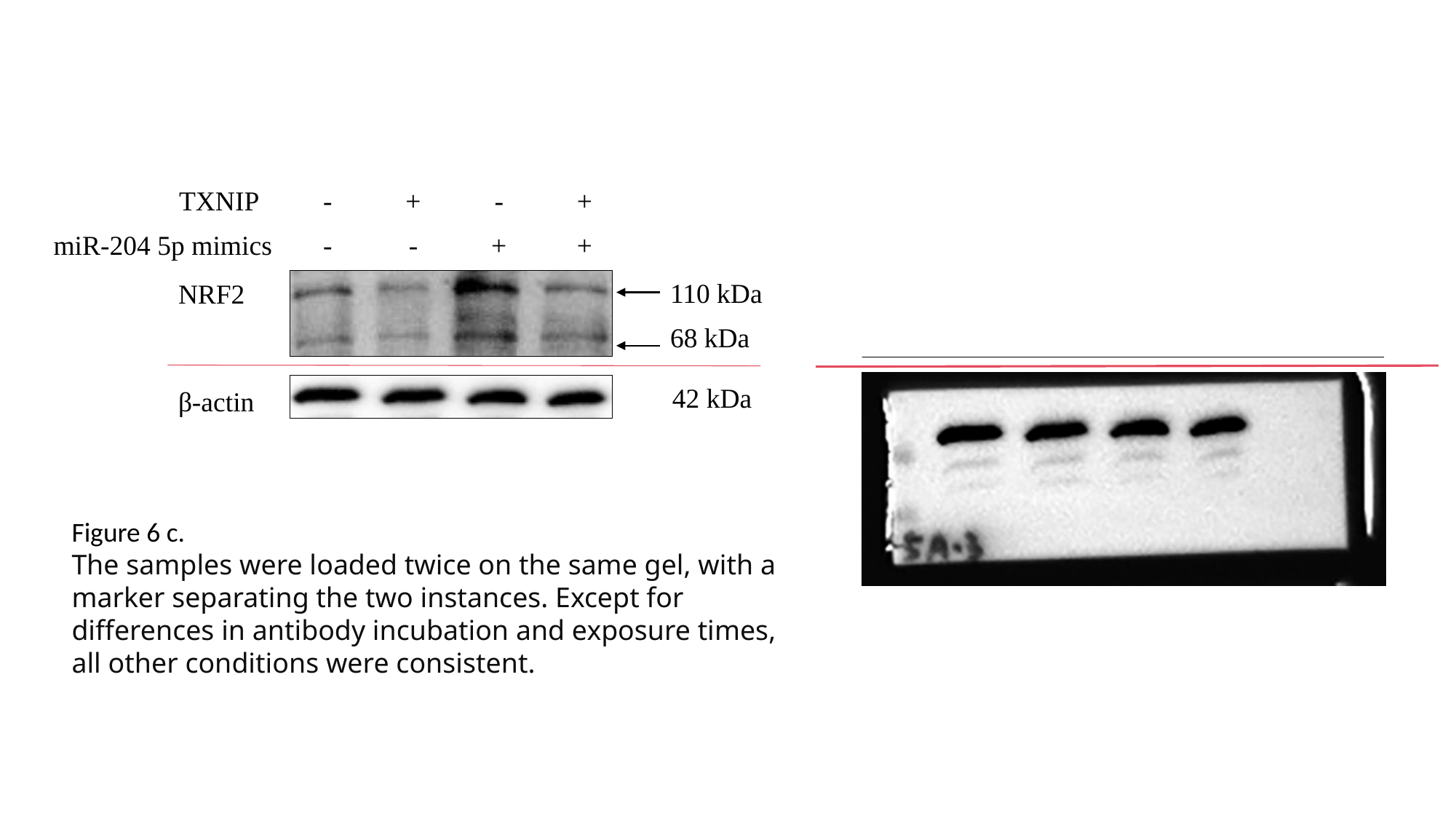

TXNIP
-
+
-
+
miR-204 5p mimics
-
-
+
+
110 kDa
NRF2
68 kDa
42 kDa
β-actin
Figure 6 c.
The samples were loaded twice on the same gel, with a marker separating the two instances. Except for differences in antibody incubation and exposure times, all other conditions were consistent.
